# Identification of a glucose-insensitive variant of Gal2 from *Saccharomyces cerevisiae* exhibiting a high pentose transport capacity

**DOI:** 10.1038/s41598-021-03822-7

**Published:** 2021-12-22

**Authors:** Sebastian A. Tamayo Rojas, Virginia Schadeweg, Ferdinand Kirchner, Eckhard Boles, Mislav Oreb

**Affiliations:** Institute of Molecular Biosciences, Faculty of Biological Sciences, Goethe University, Max-von-Laue Straße 9, 60438 Frankfurt, Germany

**Keywords:** Metabolic engineering, Carbohydrates

## Abstract

As abundant carbohydrates in renewable feedstocks, such as pectin-rich and lignocellulosic hydrolysates, the pentoses arabinose and xylose are regarded as important substrates for production of biofuels and chemicals by engineered microbial hosts. Their efficient transport across the cellular membrane is a prerequisite for economically viable fermentation processes. Thus, there is a need for transporter variants exhibiting a high transport rate of pentoses, especially in the presence of glucose, another major constituent of biomass-based feedstocks. Here, we describe a variant of the galactose permease Gal2 from *Saccharomyces cerevisiae* (Gal2^N376Y/M435I^), which is fully insensitive to competitive inhibition by glucose, but, at the same time, exhibits an improved transport capacity for xylose compared to the wildtype protein. Due to this unique property, it significantly reduces the fermentation time of a diploid industrial yeast strain engineered for efficient xylose consumption in mixed glucose/xylose media. When the N376Y/M435I mutations are introduced into a Gal2 variant resistant to glucose-induced degradation, the time necessary for the complete consumption of xylose is reduced by approximately 40%. Moreover, Gal2^N376Y/M435I^ confers improved growth of engineered yeast on arabinose. Therefore, it is a valuable addition to the toolbox necessary for valorization of complex carbohydrate mixtures.

## Introduction

Economic viability of the fermentative production of biofuels and other valuable chemicals from plant biomass, such as lignocellulosic^1^ and pectin-rich^2^ residual material, is dependent on the ability of engineered microbial cell factories to efficiently utilize all carbohydrates present in it. Besides glucose, these feedstocks contain large fractions of the pentoses xylose and/or arabinose in varying proportions depending on the biomass type. *Saccharomyces cerevisiae*, one of the most popular organisms in industrial biotechnology, is a very efficient glucose consumer, but does not have the ability to metabolize xylose and arabinose naturally. Therefore, extensive research has been devoted to expand its substrate portfolio accordingly. Overexpression of heterologous xylose isomerases (XI) proved as the most direct way to funnel xylose into the endogenous metabolism. XI converts xylose to xylulose, which can be phosphorylated by the endogenous xylulokinase, Xks1, to yield xylulose-5-P (Xu5P), an intermediate of the non-oxidative branch of the pentose phosphate pathway (noxPPP). For the conversion of arabinose to Xu5P, three bacterial enzymes must be expressed, namely L-arabinose isomerase, L-ribulokinase and L-ribulose-5-P 4-epimerase^3–5^. To improve the subsequent metabolism of Xu5P, overexpression of the noxPPP enzymes (ribose-5-phosphate ketol-isomerase, Rki; D-ribulose-5-phosphate 3-epimerase, Rpe; transketolase, Tkl; transaldolase, Tal) is necessary^6^. A combination of rational metabolic engineering and adaptive laboratory evolution approaches have resulted in great progresses in the last decade, bringing the pentose fermentation technology to the industrial scale^7,8^.

Despite these advances, the simultaneous fermentation of pentoses and glucose is still regarded as one of the major limitations of the fermentation performance^9^. Especially the ability of sugar transporters to take up pentoses in the presence of glucose is an important prerequisite for complete and time-efficient valorization of mixed-sugar substrates^9,10^. Although many endogenous^11,12^ and heterologously expressed^13^ transporters are able to take up pentoses in *S. cerevisiae*, all of them are naturally sensitive to competitive inhibition by glucose^14^. For instance, the endogenous galactose permease Gal2, which is particularly attractive due to its ability to transport both arabinose^3^ and xylose^11^, is strongly inhibited in the presence of glucose^15^. To solve this problem, several groups have successfully employed different strategies. In our previous work, we designed a screening system, based on the hexose transporter deficient strain EBY.VW4000^16^, in which all genes encoding enzymes with hexokinase (Hxk) activity have additionally been deleted. Furthermore, in this strain background, *XKS1*, the noxPPP genes *RKI1*, *RPE1*, *TAL1* and *TKL1* and a heterologous XI have been overexpressed to enable the utilization of xylose^17^. The resulting strain (AFY10X) was suitable for screening of mutant transporter variants able to transport xylose in the presence of glucose. Thereby, a single mutation of a conserved asparagine residue located in the transmembrane domain 8 (TM8) of Hxt5, Hxt7 and Gal2 was found to be crucial to alleviate the binding of glucose as a competitor^17^. Similar observations regarding the role of the conserved asparagine have also been made in further studies^18–22^ with different transporters and several mutant glucose-insensitive transporters are now available. As a rule, however, this beneficial property came at the expense of a reduced transport capacity (reflected by the kinetic parameter called maximal velocity, v_max_). The corresponding values are systematically compared in a recent review article^10^. For instance, the v_max_ for xylose transport of glucose-insensitive Gal2 variants N376F and N376V described in our previous study^17^ was approximately threefold reduced compared to the wildtype protein. In fermentations, this causes a lower net xylose consumption rate and, consequently, prolonged fermentation times. Therefore, in this study, we used the AFY10X screening platform and performed additional rounds of random mutagenesis by error-prone PCR to select novel Gal2 variants that combine glucose-insensitivity with an improved transport capacity.

## Materials and methods

### Construction and cultivation of yeast strains

The construction of the strains EBY.VW4000^16^ and AFY10^17^ was previously reported. The strain SRY027 is a derivative of the previously described HDY.GUF12^23,24^. In brief, HDY.GUF12 contains genetic cassettes for overexpression of heterologous genes necessary for the utilization of xylose (codon-optimized gene for xylose isomerase XylA from *Clostridium phytofermentans*) and arabinose (codon-optimized genes for the arabinose transporter AraT from *Scheffersomyces stipitis*, arabinose isomerase AraA from *Bacillus licheniformis*, ribulokinase AraB and ribulose-5-P 4-epimerase AraD, both from *Escherichia coli*). In addition to these heterologous genes, the open reading frames (ORFs) encoding the endogenous xylulokinase Xks1, transaldolases Tal1 and Nqm1, transketolases Tkl1 and Tkl2, Ribose-5-phosphate ketol-isomerase Rki1, D-ribulose-5-phosphate 3-epimerase Rpe1 and the sugar transporters Gal2, Hxt2, Hxt7 and Hxt9 were placed under the control of strong constitutive promoters. To construct SRY027 as a fast pentose utilizing strain for testing engineered transporters without the interference with genomic copies of Gal2, all *GAL2* alleles in HDY.GUF12 were deleted using the CRISPR technology^25^. The genotypes of all strains are listed in Table1.

**Table 1:** Strains used in this study. The genotypes are annotated according to the standard nomenclature. Promoters and terminators are denoted by suffixes “p” and “t”, respectively. Overexpressed ORFs resulting in functional proteins are underlined. The integrated genetic cassettes are shown in brackets. The occurrence of two identical alleles in a diploid genome is indicated by “2×”. Gene deletions are indicated by a “Δ”, followed by the range of deleted amino acids, where appropriate. Parental strains are indicated in bold under “relevant genotype”.

Strain name	Relevant genotype	References
CEN.PK2-1C	*leu2-3,112 ura3-52 trp1-289 his3Δ1 MAL2-8c SUC2*	Euroscarf
EBY.VW4000	**CEN.PK2-1C** *Δhxt1-17 gal2Δ::loxP stl1Δ::loxP agt1Δ::loxP mph2Δ::loxP mph3Δ::loxP*	Ref.^16^
AFY10	**EBY.VW4000** *glk1Δ::loxP hxk1Δ::loxP hxk2Δ::loxP ylr446wΔ::loxP* [*pyk2Δ::PGK1p-opt.XKS1-PGK1t TPI1p-TAL1-TAL1t TDH3p-TKL1-TKL1t PFK1p-RPE1-RPE1t FBAp-RKI1-RKI1t kanMX*]	Ref.^17^
AFY10X	**AFY10** with YEp181-kanR_optXI plasmid	Ref.^17^
SRY027	**Ethanol Red** (diploid) *MATα/MATa;* 2 × [*pyk2Δ::PGI1p-araA-PGI1t, PYK1p-araD-PYK1t, PGM2p-araB-PGM2t, PFK2p-ARAT-PFK2t, PMA1p-NQM1-ZWF1t, ADH1p-TKL2-PDC1t, loxP, GPM1p-HXT7-HXT7t, FBA1p-RKI1-RKI1t, PFK1p-RPE1-RPE1t, TDH3p-TKL1-TKL1t, TPI1p-TAL1-TAL1t, PGK1p-XKS1-PGK1t, HXT7p*^*-1–392*^*-xylA-CYC1t*]; 2 × [*HXT7p*^*-1–392*^*-xylA-CYC1t, PGM2p-araB-PGM2t, PYK1p-araD-PYK1t, PGI1p-araA-PGI1t, GAL2p::FBA1p-GAL2*^*Δ70–1655*^*-GAL2t*]; 2 × [*HXT7p*^*-1–392*^*-xylA-CYC1t, PYK1p-HXT9-HXT9t, PGI1p-araA-PGI1t, HXT2p::pENO2-HXT2*]	This study

For the preparation of competent cells, plasmid-free cells were grown in standard YEP-media (1% (w/v) yeast extract, 2% (w/v) peptone) supplemented with 1% (w/v) maltose (M) for EBY.VW4000, 2% (v/v) ethanol (E) for AFY10 and 2% (w/v) of glucose (D) for SRY027. Frozen competent cells were prepared and transformed according to the established protocol^26^. The transformants of auxotrophic strains were initially plated on solid, selective synthetic complete (SC) medium with 1% (w/v) maltose (EBY.VW4000) or 2% (v/v) ethanol (AFY10), in which uracil (-Ura), leucine (-Leu), histidine (-His) and tryptophan (-Trp) were omitted as required depending on the plasmid selection markers. Amino acids were added as previously described^27^. SRY027 was plated on YEPD medium supplemented with 100 µg mL^−1^ ClonNAT (Nourseothricin) or 200 µg mL^−1^ G418 (Geneticin) for the selection of the *natNT2* and *kanMX* markers, respectively.

### Plasmid construction and mutagenesis approaches

The DNA sequence of the *GAL2* ORF that was used for plasmid construction and mutagenesis approaches corresponds to CEN.PK2-1C variant. For error-prone PCR (epPCR), the GeneMorph II Random Mutagenesis Kit (Agilent Technologies) was used as previously described^17^. Briefly, the mutagenesis reaction was performed with the primer pair Amp_GAL2_F/R using the p426H7_GAL2 plasmid as template. The epPCR fragments were purified by extraction from agarose gels (NucleoSpin Extract II; Macherey–Nagel) and subjected to a non-mutagenic second round of PCR using the Phusion polymerase (New England Biolabs GmbH) with the primer pair Clon_GAL2_F/R to add overhangs for homologous recombination with the p426H7 vector. AFY10X was transformed with the linearized p426H7 vector and the epPCR products and plated onto selective SCE plates. After colonies appeared, they were replica-plated to plates with various xylose–glucose concentrations (2 + 10, 2 + 20, 10 + 30, and 10 + 50 g L^−1^) and screened for the fastest growing clones. Site directed mutagenesis was performed using mutagenic primers listed in Supplementary Table 1 and the Phusion polymerase. Green fluorescent protein (GFP) was fused to the C-terminus of Gal2 variants as described previously^28^. Plasmids were isolated from yeast by the standard alkaline lysis protocol and electroporated into *E. coli* strain DH10B for amplification. Plasmid isolation from overnight *E. coli* cultures was carried out using a GeneJET Plasmid Miniprep Kit (Thermo Scientific) according to the manufacturer’s instructions and sequenced. The plasmids used in this study are listed in Table 2.

**Table 2:** Plasmids used in this study**.** Under “relevant properties”, promoters and terminators are denoted by suffixes “p” and “t”, respectively; ORF, open reading frame.

Plasmid name	Relevant properties	References
YEp181-kanR_optXI	2μ, *LEU2*, codon-optimized xylose isomerase gene *xylA* of *C. phytofermentans* under control of *HXT7p*^*-1-392*^ and *CYC1t*, Ampicillin marker	Ref.^15^
p426H7	2μ, *URA3*, *HXT7p*^*-1-392*^*, CYC1t*, Ampicillin marker, pBR322-origin	Ref.^11^
p426H7_GAL2_WT	ORF of GAL2 in p426H7	Ref.^17^
p426H7_GAL2_ep3.1	ORF of GAL2-M107K/V239L/N376Y/M435I/L558S in p426H7	This study
p426H7_GAL2-N376Y	ORF of GAL2-N376Y in p426H7	Ref.^17^
p426H7_GAL2-N376Y/M107K	ORF of GAL2-N376Y/M107K in p426H7	This study
p426H7_GAL2-N376Y/V239L	ORF of GAL2-N376Y/V239L in p426H7	This study
p426H7_GAL2-N376Y/M435I	ORF of GAL2-N376Y/M435I in p426H7	This study
p426H7_GAL2-N376Y/M558S	ORF of GAL2-N376Y/M558S in p426H7	This study
p426H7_GAL2-M435I	ORF of GAL2-M435I in p426H7	This study
p426H7_GAL2-N376F	ORF of GAL2-N376F in p426H7	Ref.^17^
p426H7_GAL2-N376F/M435I	ORF of GAL2-N376F/M435I in p426H7	This study
pRS62N	2μ, *natNT2*; *HXT7p*^*-1-392*^, *CYC1t*, Ampicillin marker, pBR322-origin	Ref.^17^
pRS62N_Gal2_WT	ORF of GAL2 in pRS62N	Ref.^17^
pRS62N_Gal2_N376F	ORF of GAL2-N376F in pRS62N	Ref.^17^
pRS62N_Gal2_N376F/M435I	ORF of GAL2-N376F/M435I in pRS62N	This study
pRS62N_Gal2_N376Y/M435I	ORF of GAL2-N376Y/M435I in pRS62N	This study
pRS62N_Gal2_6SA/N376Y/M435I	ORF of Gal2_6SA/N376Y/M435I in pRS62N	This study
pRS62N_Gal2_6SA	ORF of Gal2_6SA in pRS62N	This study
pRCC-K	2μ, *kanMX4* marker, employed for the CRISPR/Cas9 deletions; *ROX3p*-cas9-*CYC1t*; *SNR52p-gRNA-SUP4t*	Ref.^25^
pRCC-K_GAL2	pRCC-K with a protospacer targeting *GAL2*	This study
YEparaAsynth	2μ, *HIS3*, codon-optimized *araA* gene from *Bacillus licheniformis*, expressed under control of *HXT7p*^*-1—392*^ and CYC1t, Ampicillin marker, pBR322-origin	Ref.^5^
YEparaBsynth	2μ, *TRP1*, codon-optimized *E. coli* araB, expressed under control of *HXT7p*^*-1-392*^ and *CYC1t*, Ampicillin marker, pBR322-origin	Ref.^5^
YEparaDsynth	2μ, *LEU2*, codon-optimized *E. coli araD*, expressed under control of *HXT7p*^*-1-392*^ and *CYC1t*, Ampicillin marker, pBR322-origin	Ref.^5^
pUCP1	*CEN6/ARS4*, *URA3*, Ampicillin marker, *HXT7p*^*-1-392*^*, CYC1t*	Ref.^28^
pUCP1 GAL2^N376Y/M435I^-GFP	pUCP1 *GAL2*^*N376*^^*Y/M435I*^*-GFP* under control of *HXT7p*^*-1-392*^ and *CYC1t*	This study
pUCP1 GAL2^6^^SA/N376Y/M435I^-GFP	pUCP1 *GAL2*^*6SA/N376Y/M435I*^*-GFP* under control of *HXT7p*^*-1-392*^ and *CYC1t*	This study

### Fermentation experiments and HPLC analysis

Cells were inoculated into selective media as indicated in the Results. Fermentations were performed in 300 mL shake flasks containing 50 mL of the culture medium. The cultures were cultivated at 30 °C with agitation at 180 rpm. For HPLC analysis, 450 µL of the culture supernatant were mixed with 50 µL 50% (w/v) 5-sulfosalicylic acid and centrifuged (16,000 g, 5 min, 4 °C). From this supernatant, 10 µL were subjected to high performance liquid chromatography (Dionex Ultimate 3000, Thermo Scientific) equipped with the ion exchange column HyperREZ XP Carbohydrate H^+^ (7.7 × 300 mm; Thermo Scientific) and a refractive index detector. The liquid phase was 5 mM sulfuric acid, run at 0.6 ml min^−1^ and 65 °C. The Chromeleon 6.8 software was employed for quantifications and Prism 9 (GraphPad) for statistical analysis and presentation of the results.

### Radiolabeled xylose uptake assay

The uptake assay with radiolabeled xylose was performed according to the published protocol^29^. In brief, EBY.VW4000 yeast cells expressing the indicated Gal2 variants were grown overnight at 30 °C and 180 rpm in SCM-Ura medium. The next day, cells were harvested by centrifugation (4000 g, 5 min, 20 °C). The pellets were washed twice in ice-cold 0.1 M potassium phosphate buffer (pH 6.5), weighed, and resuspended in 0.1 M potassium phosphate buffer to a wet-weight concentration of 60 mg ml^−1^. Aliquots of 110 µl were prepared and cooled on ice. The residual cell suspension was centrifuged (4,000 g, 5 min, 4 °C) and the pellet was stored at − 80 °C before it was freeze-dried for cell dry weight determination. D-[1-^14^C] xylose (55 mCi mmol^−1^) was obtained from American Radiolabeled Chemicals Inc. (St. Louis, MO) and diluted with unlabeled sugar to approximately 8 nCi mmol^−1^ and a total xylose concentration of 1500 mM (3 × concentration of that needed in the final uptake mixture). The cell suspension aliquots and the sugar solution were pre-warmed at 30 °C for 5 min. The uptake reaction was started by mixing 100 µl of the cell suspension and 50 µl of the 3 × sugar solution (yielding 500 mM total xylose in the uptake assay). The mixture was incubated for 20 s at 30 °C. The uptake was terminated by pipetting 100 µl of the assay mixture into 10 ml of ice-cold uptake buffer containing 500 mM xylose (quenching buffer). The suspension was filtered through a PVDF membrane filter, 0.22 µM pore size, 47 mm diameter (Durapore, Merck), which was subsequently washed two times with 10 ml of ice-cold quenching buffer. The amount of the radiolabeled sugar was measured in a 10 min window using the Wallac 1409 Liquid Scintillation Counter (Perkin Elmer). The proportion of the intracellular sugar was calculated as a ratio of the counts on the filter (corrected for background binding) to total counts in the uptake mixture. The transport rate (nmol min^−1^) was calculated and normalized to the dry cell weight.

### Fluorescence microscopy

For fluorescence microscopy, CEN.PK2–1C cells expressing the GFP-tagged constructs were cultivated overnight in 50 mL filter-sterilized, low fluorescent SC medium with 3% (w/v) glucose and 1% (w/v) xylose lacking uracil (lf-SC-Ura), containing 6.9 g/L yeast nitrogen base with ammonium sulfate, without amino acids, without folic acid and without riboflavin (MP Biomedicals, Santa Ana, CA). Subsequently, the cells were washed twice with sterile water and re-suspended in 50 mL of fresh medium to an OD_600_ between 1.5 and 3. From these cultures, after 4 h of cultivation at 30 °C with shaking (180 rpm), an aliquot of each one was mixed separately with 500 μL of medium containing 1.2% (w/v) low melting agarose (Roth) to reach a final suspension of 0.6% (w/v) low melting agarose for immobilization of cells. The final cell density in the suspension was OD_600_ ≈ 2. A total of 6 μL of it were applied to an object plate and sealed with a cover slip. GFP fluorescence was detected with the Confocal Laser Scanning Microscope Zeiss LSM 780 (Jena, Germany).

### Homology modeling

The model of Gal2 was created with SWISS-MODEL (https://swissmodel.expasy.org)^30^ using the crystal structure of XylE (PDB ID: 4GBZ)^31^ as a template. The localization of glucose in the substrate binding pocket of Gal2 was inferred by superimposing the model with the glucose-bound XylE structure. The amino acid replacement and visualization were performed with YASARA View^32^, version 17.4.17.

## Results

### Identification of improved Gal2 mutants

To identify Gal2 variants with improved xylose transport properties in the presence of glucose, the *GAL2* wildtype open reading frame was subjected to error-prone PCR (epPCR) as described in “Materials and methods”. The obtained PCR products were co-transformed with the linearized p426H7 plasmids into the strain AFY10X to allow for in vivo plasmid assembly and the transformants were initially plated on the permissive, ethanol-containing (SCE-Ura) agar plates. The resulting colonies were subsequently replica-plated on media containing glucose/xylose mixtures and screened for fastest growth based on the colony size. One of fastest growing clones (designated ep3.1) was selected for further characterization. The *GAL2*-plasmid was isolated and sequenced, revealing five distinct mutations causing amino acid exchanges, namely M107K, V239L, N376Y, M435I and L558S. The substitution N376Y was already described in our previous publication to enable glucose-insensitive xylose transport, but its transport activity for the pentose was rather low^17^. We therefore concluded that at least one of the additional mutations is responsible for the improved phenotype of the ep3.1 mutant. To test this assumption, we combined each of the four mutations individually with N376Y and tested the growth conferred by the resulting plasmids on different carbon sources. For growth tests on hexoses (glucose and galactose) the strain EBY.VW4000, which is deficient for all transporters capable of hexose transport in *S. cerevisiae*^16^ was used, whereas the growth on pentoses was assayed in the screening strain AFY10X. In accordance with our previous observations^17^, the constructs containing the N376Y mutations were not able to confer growth on glucose (Supplementary Fig. S1). On xylose or xylose/glucose mixtures, the superior properties of the ep3.1 variant compared to the N376Y single mutant could be clearly attributed to the M435I mutation (Supplementary Fig. S2). The N376Y/M435I variant was the only double mutant that conferred strong growth on pure xylose and glucose/xylose mixture.

**Figure 1:**
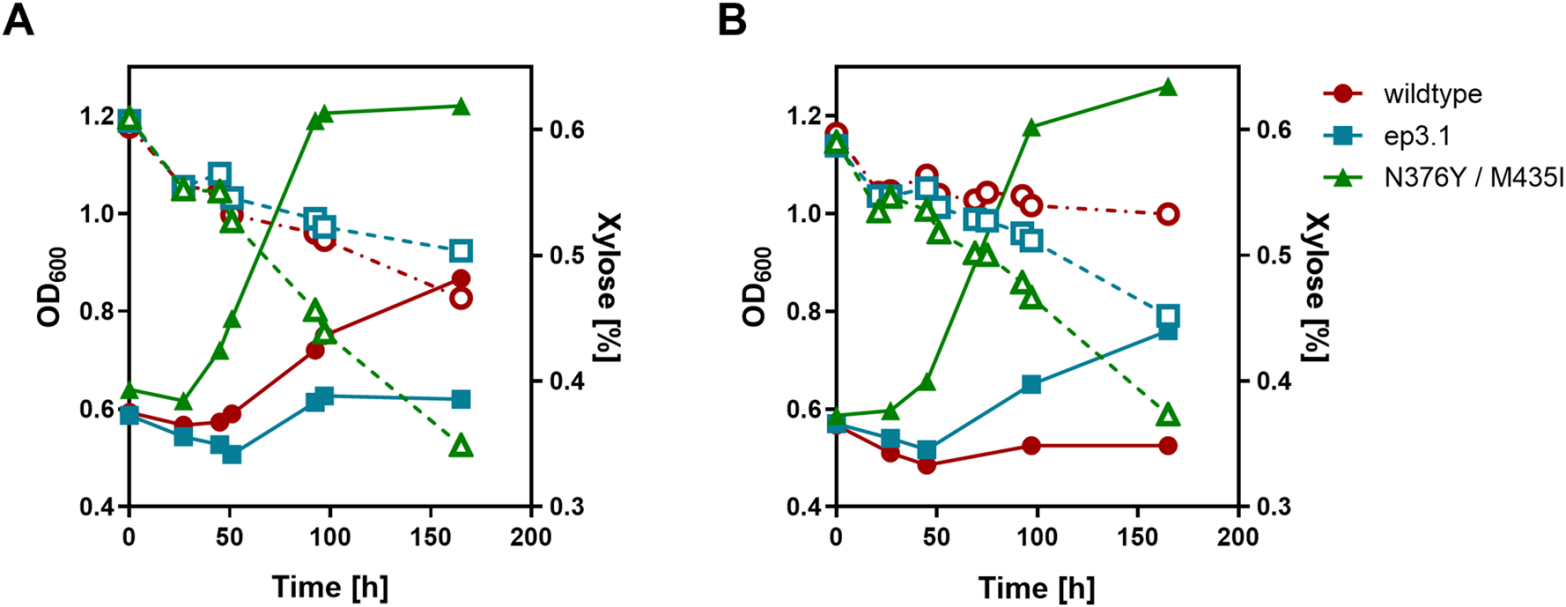
Fermentation of the screening strain AFY10X transformed with different Gal2 variants. The Gal2 constructs (wildtype, the selected mutant ep3.1 and the N376Y/M435I double mutant) were expressed from multicopy plasmids in the hxt^0^/hxk^0^ strain AFY10X. The consumption of xylose (0.5% w/v; open symbols, dashed lines) in the absence (**A**) or presence (**B**) of glucose (2% w/v) and growth curves (OD_600_; closed symbols, full lines) are shown. The mean values of three independent cultures are shown. The error bars are omitted for clarity.

To see more subtle differences between the different variants than is possible on agar plates, we grew liquid cultures and measured the consumption of xylose in AFY10X cells transformed with the wildtype, ep3.1 and the N376Y/M435I variants (Fig. 1). The media contained 0.5% (w/v) xylose with or without glucose addition (2% w/v). Comparing the growth and xylose consumption rates, the N376Y/M435I variant performed even better than the ep3.1 construct, suggesting that at least one of the remaining ep3.1 mutations (M107K, V239L and L558S) has a negative impact on the xylose transport activity.

Interestingly, the M435I mutation alone appears to have a negative influence on the transport of both glucose and xylose (Supplementary Fig. S3), which implies that there is a synergistic effect of the N376Y and M435I mutations in improving the uptake of xylose. We therefore also combined M435I with N376F, which has a lower v_max_ for xylose compared to wildtype Gal2^17^. In growth tests on agar plates containing pure xylose or a mixture of xylose and glucose, the N376F/M435I variant performed comparably to the N376F single mutant, but slightly worse than the N376Y/M435I double mutant (Supplementary Fig. S4).

### Xylose uptake capacity of mutant Gal2 variants

**Figure 2:**
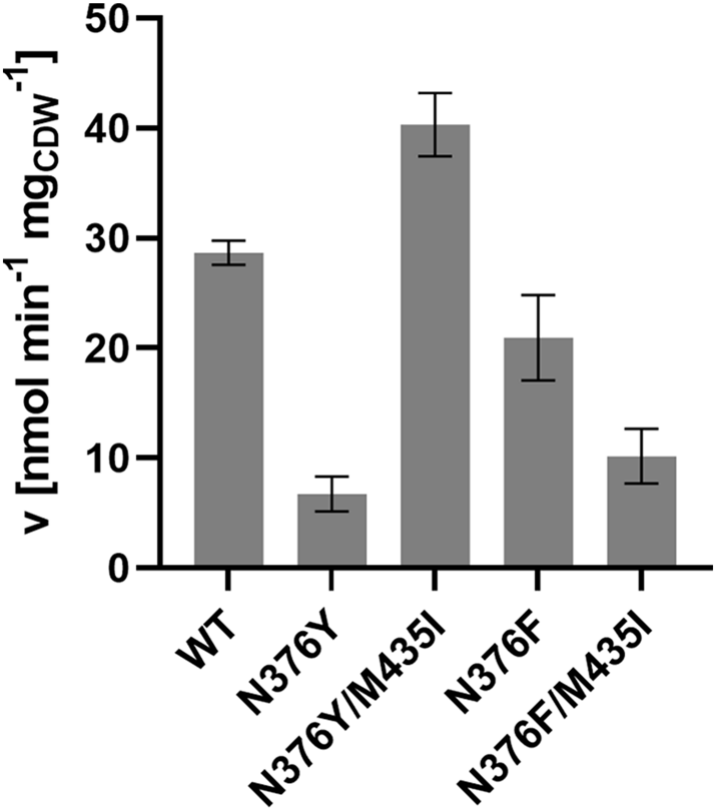
Uptake of ^14^C-labelled xylose by selected Gal2 variants. Gal2 wildtype (WT) and indicated mutants were expressed in the hxt^0^ strain EBY.VW4000 and the uptake velocity was measured at 500 mM total xylose. The uptake velocity is shown as nmol xylose taken up per minute per mg cell dry weight (nmol min^−1^ mg_CDW_^−1^). Mean values and standard deviation of triplicate measurements are shown.

To better understand the results described above and to analyze the transport activity of the Gal2 variants independently of cell growth, which is influenced by multiple factors, we performed sugar uptake assays with radiolabeled (^14^C) xylose (Fig. 2). To estimate the transport capacity (defined by the maximum velocity, v_max_), the assays were performed at nearly saturating xylose concentrations (500 mM). Strikingly, the measured transport velocities mirror the growth behavior of the transformants described above. Whereas the N376Y mutation alone negatively affects xylose transport^17^ (see also Supplementary Fig. S2), this defect is more than compensated by the additional M435I mutation. The lower transport rate by the N376F and N376F/M435I variants is consistent with the results shown in Supplementary Fig. S4.

Taken together, the results show that the M435I substitution increases the transport capacity in combination with tyrosine (but not phenylalanine) at position 376 in a synergistic manner.

### Fermentation performance of engineered strains expressing mutant Gal2 variants

**Figure 3:**
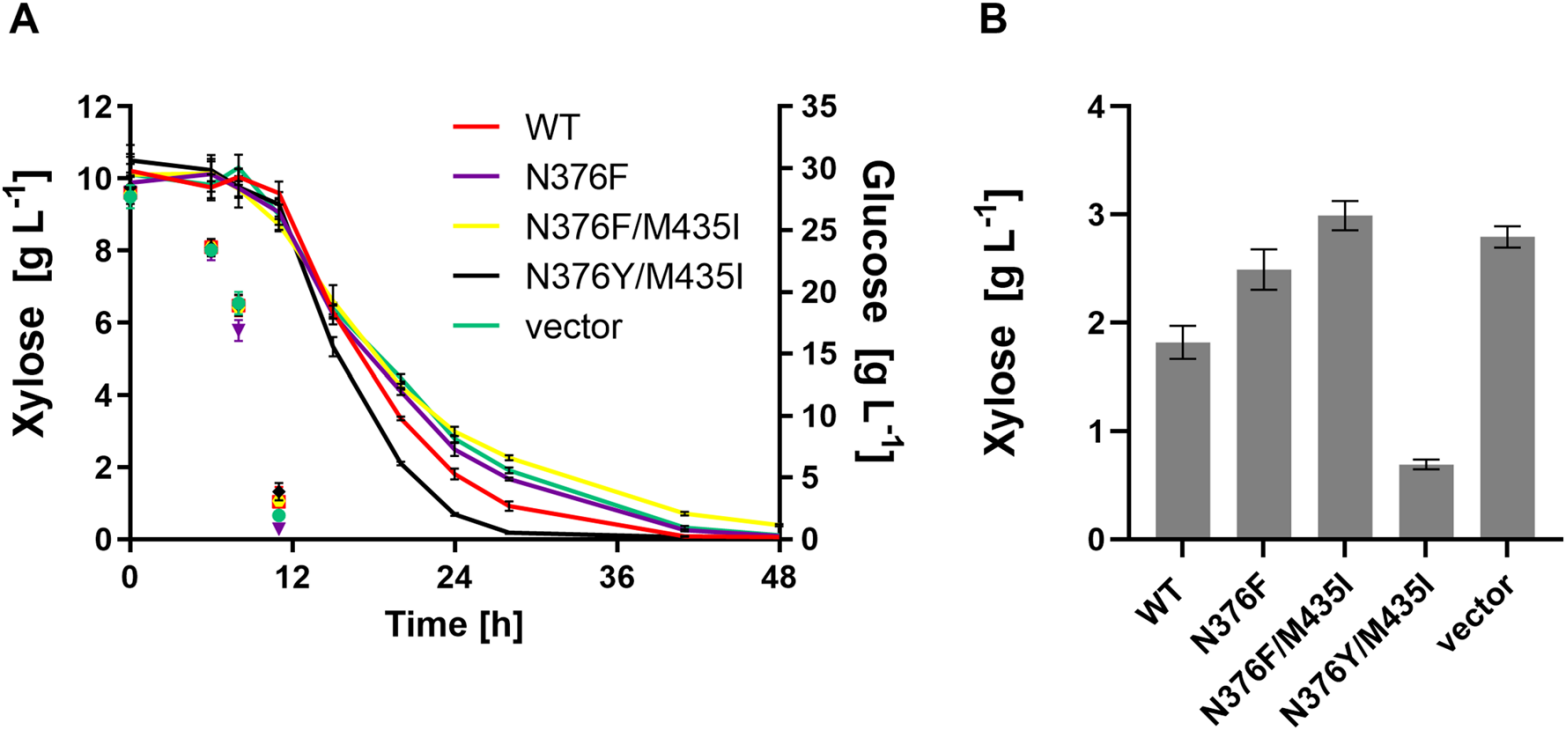
Fermentation of the industrial strain SRY027 expressing different Gal2 variants. The Gal2 constructs (wildtype—WT or the indicated mutants) were expressed from multicopy plasmids in the strain SRY027. (**A**) The consumption of xylose (starting concentration 10 g L^−1^, solid lines; plotted on the left Y-axis) and glucose (starting concentration 30 g L^−1^; symbols only, plotted on the right Y-axis) was measured via HPLC analysis. The mean values and standard deviation of three independent cultures are shown. The error bars may be smaller than the symbols. (**B**) Residual xylose concentration measured after 24 h of fermentation is shown. All values differ significantly from the wildtype (Tukey's multiple comparisons test, *P* < 0.01).

After having demonstrated the superior properties of the N376Y/M435I mutant in the screening strain AFY10X, which consumes xylose very slowly (see Fig. 1), we reasoned that the “true” utility of the new transporter variant for mixed-sugar fermentations could be best challenged in a system highly optimized for pentose fermentation. We selected the strain SRY027 (see “Materials and methods” and Table 1 for details), derived from the robust, diploid industrial strain HDY.GUF12 (Ethanol Red background), which was genetically modified by integrating overexpression cassettes necessary for xylose and arabinose utilization, and further optimized to consume xylose through adaptive laboratory evolution^23,24^. In SRY027, the genomic *GAL2* alleles were deleted to avoid any interferences with the engineered *GAL2* constructs. In this background, we overexpressed different Gal2 variants from a multicopy (2µ) plasmid and performed fermentations in glucose/xylose mixtures (30 g L^−1^ and 10 g L^−1^, respectively). All transformants consumed the sugars sequentially, with a fast glucose consumption phase (up to 12 h), followed by a slower, albeit considerably efficient xylose utilization, where the pentose sugar was consumed within approximately 36 h. Despite the presence of glucose-insensitive transporter variants (all mutants in which N376 was substituted by either Y or F), none of the transformants was able to significantly co-consume both sugars. Nevertheless, they did differ in the rate of xylose consumption (Fig. 3) in a manner that mirrors the xylose uptake capacities shown in Fig. 2, i.e. the N376Y/M435I exhibiting the highest and N376F/M435I the lowest velocity.

**Figure 4:**
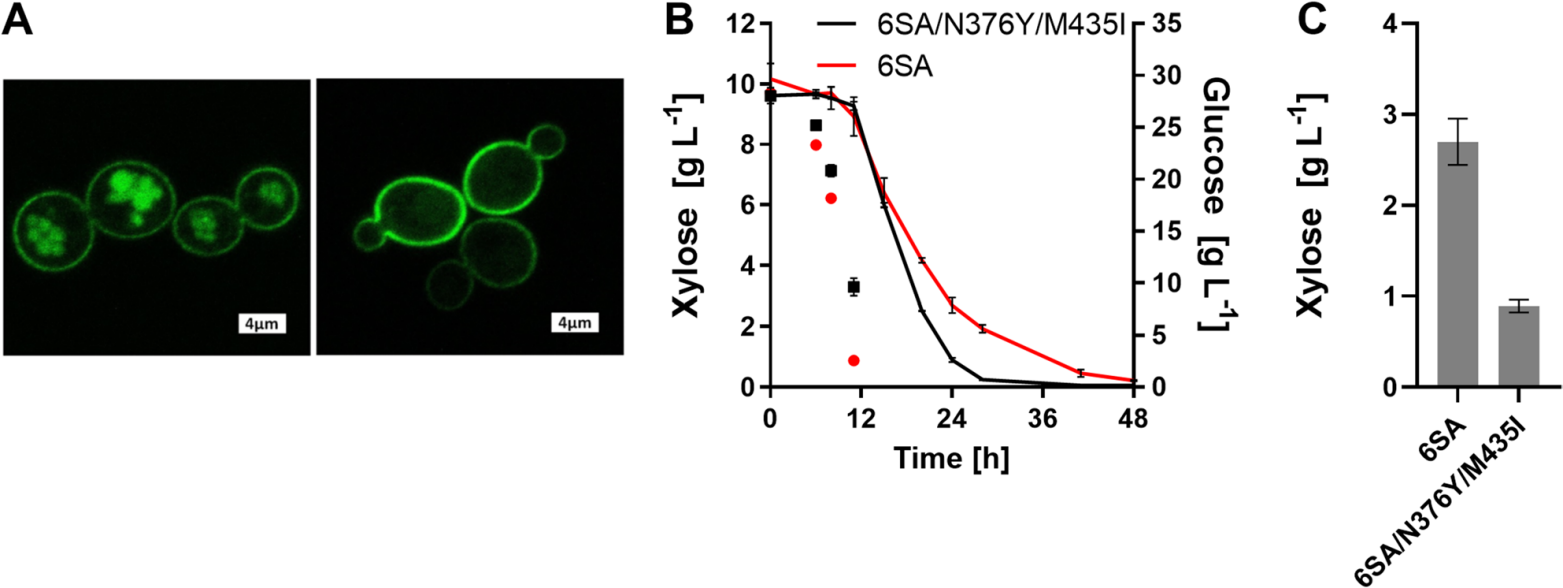
Stabilization of Gal2 variants by the 6SA N-terminal tail. The sextuple mutation within the N-terminal tail (6SA) conferring resistance to glucose-induced internalization was combined with the indicated Gal2 variants. (**A**) N376Y/M435I (left) and 6SA/N376Y/M435I (right) Gal2 variants were expressed as GFP fusions from single copy plasmids in CEN.PK2-1C and the localization of the transporters was analyzed by fluorescence microscopy. (**B**) The consumption of xylose (starting concentration 10 g L^−1^, solid lines; plotted on the left Y axis) and glucose (starting concentration 30 g L^−1^; symbols only, plotted on the right Y axis) was measured via HPLC analysis. The mean values and standard deviation of three independent cultures are shown. The error bars may be smaller than the symbols. (**C**) Residual xylose concentration measured after 24 h of fermentation is shown. The values differ significantly (unpaired two-tailed *t* test, *P* < 0.01). The fermentation experiments in (**B**) and (**C**) were performed with the strain SRY027, in which the Gal2 constructs were expressed from multicopy plasmids.

One possible explanation for the inability of the cells to co-consume glucose and xylose could be the glucose-induced internalization and degradation of Gal2 in the vacuole^33^. This process is induced by phosphorylation and ubiquitination of the N-terminal cytoplasmic tail and can be abolished by mutating six serine residues at positions 32, 35, 39, 48, 53 and 55 to alanine. The resulting Gal2^6SA^ is stable at the plasma membrane even in the presence of glucose^28^. Therefore, we combined the 6SA N-terminal tail with the N376Y/M435I double mutation. To investigate if the 6SA tail is stabilizing the N376Y/M435I mutant, we also generated green florescent protein (GFP) fusion constructs as previously described^28^. As shown by fluorescence microscopy, the N376Y/M435I variant is internalized in glucose/xylose mixtures, whereas the 6SA/N376Y/M435I is nearly exclusively localized at the plasma membrane (Fig. 4A), as expected. Therefore, we transformed the stabilized constructs (without fused GFP) into the SRY027 strain, and performed a fermentation experiment in mixed-sugar medium. Again, the N376Y/M435I variant enabled faster xylose consumption compared to the control (Fig. 4B,C), reducing the overall duration of total xylose consumption by approximately 20 h (corresponding to 40% of the total fermentation time). Still, the consumption of glucose and xylose was largely sequential. This suggests that the reason for the inability of the strain for the simultaneous fermentation of both sugars does not primarily lie at the level of transport but might be rather due to metabolic constraints (see “Discussion”).

### The N376Y/M435I variant confers improved growth on arabinose as well

**Figure 5:**
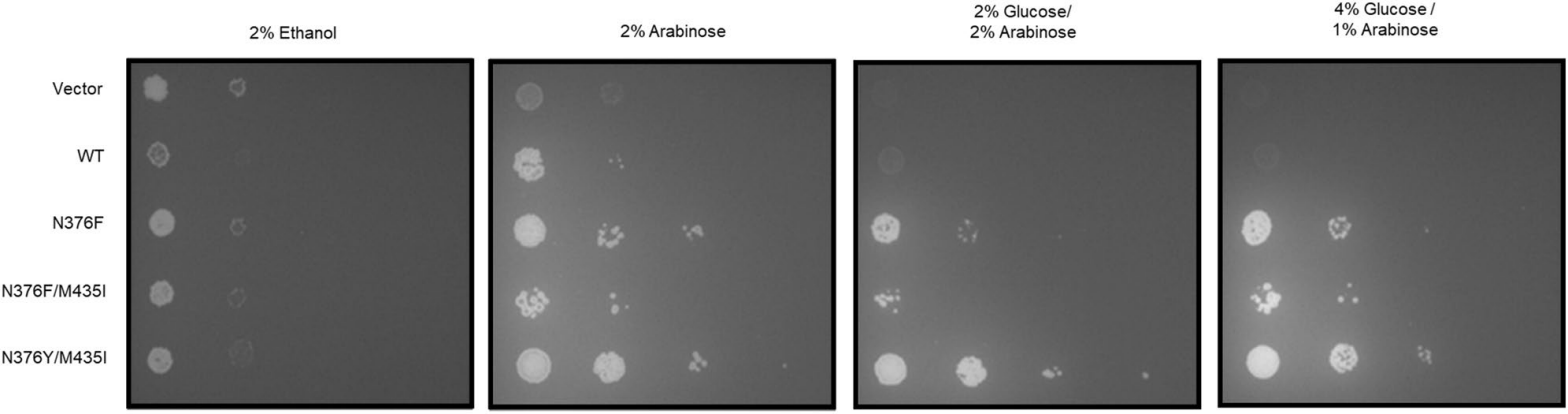
Growth conferred by different Gal2 variants on arabinose-containing media. The strain AFY10 was transformed with 2μ plasmids expressing the indicated Gal2 variants in combination with three additional 2μ plasmid expressing araA, araB and araD. The transformants were pre-grown in liquid selective SC medium with ethanol as a permissive carbon source and spotted onto selective SC solid medium containing the indicated carbon sources. Cells were grown at 30 °C for 6 days.

Besides xylose, arabinose is a relevant constituent of plant biomass. Since Gal2 is well-known to transport arabinose, we wanted to test if the N376Y/M435I double mutation is beneficial for the utilization of this pentose as well. AFY10 was enabled to metabolize arabinose by transforming it with plasmids encoding L-arabinose isomerase (*araA*), L-ribulokinase (*araB*), L-ribulose-5-P 4-epimerase (*araD*) and different Gal2 variants (wildtype, N376F, N376F/M435I and N376Y/M435I). Growth tests were performed on solid media containing arabinose or arabinose/glucose mixtures with these transformants. Expectedly, all N376 mutants conferred glucose-resistant growth on arabinose. Moreover, on all media, the N376Y/M435I variant conferred the strongest growth (Fig. 5), similar to what was observed with xylose (see Supplementary Fig. S4). This demonstrates that the Gal2^N376^^Y/M435I^ could also be used to improve the fermentation of hydrolysates that are rich in arabinose and glucose, such as pectin-rich biomass hydrolysates.

## Discussion

Here, we isolated an improved variant of Gal2 (N376Y/M435I), which combines two properties highly desirable for fermentations of lignocellulosic or pectin-rich hydrolysates – the ability to transport pentoses in the presence of glucose (Figs., 1B, 5) and an improved capacity for xylose (Figs. 1A, 2, 3, 4) and arabinose (Fig. 5) transport. Interestingly, both N376Y^17^ (Fig. 2) and M435I (Supplementary Fig. 3) mutations individually have a negative impact on xylose transport, which is more than compensated when they are combined (Fig. 2). Strikingly, another substitution at position 435 was identified among multiple mutations in a Gal2 variant that had been generated by error prone PCR and selected for growth on low xylose concentration^34^. The authors did not analyze the individual contributions of the four mutations (L301R, K310R, N314D, M435T), but considering our results it is likely that M435T is at least partly responsible for the observed phenotype, possibly in a synergistic manner, as described here. In a screen aiming to identify hexose transporter variants with increased specificity towards xylose, Zhao and coworkers^35^ isolated a double mutant of Hxt2 (T339P/M420I, where M420 corresponds to M435 of Gal2). Although this variant was not glucose-insensitive, its specificity (calculated as a ratio of the K_M_ values for the respective sugar) was shifted from glucose to xylose compared to the wildtype and the mutations acted in a synergistic manner. These reports demonstrate the importance of the conserved methionine residue corresponding to M435 of Gal2 in a different experimental context.

**Figure 6:**
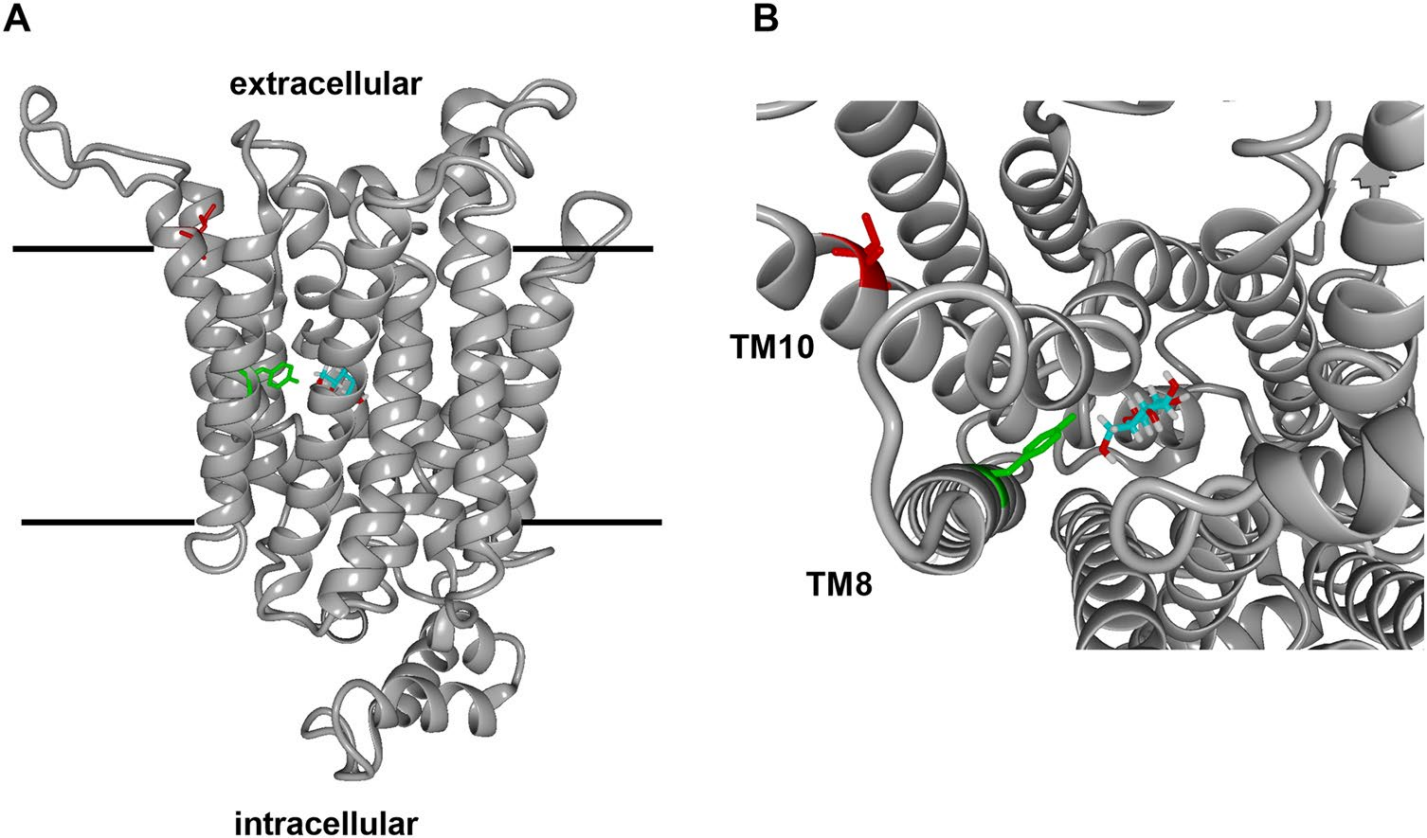
Homology model of the Gal2^N376^^Y/M435I^ mutant. The model of membrane-inserted Gal2^N376Y/M435I^ is shown as ribbon diagram in (**A**) with bound glucose (cyan backbone) and side chains of Y376 (green) and I435 (red). The view from the extracellular side (**B**) shows the positioning of TM8 and TM10, which could be affected by both mutations. The model was generated using SWISS-MODEL based on the crystal structure of XylE (PDB ID: 4GBZ) and visualized with YASARA View.

Since no crystal structure of Gal2 is available, it is only possible to speculate about the underlying mechanism on the basis of homology models. The residue 435 is located at the periphery of the transmembrane helix 10 (TM10), at the interface of the lipid bilayer and the extracellular space. Hence, it is distant from the residue 376 that is located close to the sugar binding pocket and decisive for discrimination between pentoses and hexoses (Fig. 6A). The introduction of tyrosine, a large hydrophobic residue at the position 376 instead of asparagine in TM8 could cause clashes and affect the positioning of the proximal TM10. In turn, substituting methionine at the position 435 by the more hydrophobic isoleucine could positively influence the mobility of TM10 within the lipid bilayer at the extracellular membrane interface and thereby alleviate the possible steric constraints introduced by N376Y (Fig. 6B).

It was previously postulated that the sequential consumption of glucose and xylose is mainly due to the competition of both sugars at the level of sugar transporters, which have a much higher affinity for the hexose^15^. Our results show that the expression of glucose-insensitive transporters (Fig. 3), even in an internalization-resistant form (Fig. 4) is not sufficient to enable simultaneous consumption of both sugars. This is consistent with more recent work showing that the interferences of glucose and xylose are more complex and also involve their metabolism as well as glucose repression mechanisms. For instance, laboratory evolution for forced glucose-xylose co-consumption revealed mutations affecting the activity of the hexokinase Hxk2, the Gal83 subunit of the Snf1 kinase complex and of the E3-ubiquitin ligase Rsp5^36^. In another study, it was demonstrated that reducing the hexokinase activity is crucial for achieving simultaneous consumption of glucose and xylose^37^. Interestingly, a reduction in the xylose consumption rate was also observed during the co-consumption of maltose^15^, a disaccharide that does not interfere with xylose transport, but is intracellularly cleaved into two glucose moieties, which are subsequently phosphorylated by hexokinases and thereby enter glycolysis. These observations collectively suggest that a fast glucose metabolism (and related signaling) impede the simultaneous consumption of pentoses, but the details of the underlying mechanism are still not well understood. It is conceivable that a buildup of the noxPPP intermediates derived from the fast glucose metabolism prevent the entrance of xylose (via Xu5P) into the equilibrium-driven “scrambling” reactions of this pathway. However, many other metabolic and signaling interferences are possible and further research is necessary to disentangle the individual contributing components.

In conclusion, although Gal2^N376^^Y/M435I^ alone does not facilitate co-fermentation of glucose and pentoses, it does significantly reduce the overall fermentation time and therefore should prove valuable for valorization of substrates, which contain mixtures of glucose, xylose and/or arabinose, such as lignocellulosic or pectin-rich biomass.

## Supplementary Information

Supplementary Information.
