# Supplementary material for: Identification of a glucose-insensitive variant of Gal2 from Saccharomyces cerevisiae exhibiting a high pentose transport capacity
Source: Sci Rep. 2021 Dec 22;11:24404. doi: 10.1038/s41598-021-03822-7 (PMC8695581; doi:10.1038/s41598-021-03822-7)
Supplement: Supplementary file 1 — Supplementary Information. [file 41598_2021_3822_MOESM1_ESM.pdf]

Supplementary Information

Identification of a glucose-insensitive variant of Gal2 from *Saccharomyces cerevisiae* exhibiting a high pentose transport capacity

Sebastian A. Tamayo Rojas, Virginia Schadeweg, Ferdinand Kirchner, Eckhard Boles* and Mislav Oreb*

Institute of Molecular Biosciences, Faculty of Biological Sciences, Goethe University Frankfurt, Frankfurt am Main, Germany

*Corresponding authors

Eckhard Boles

Goethe University Frankfurt

Institute of Molecular Biosciences

Max-von-Laue Straße 9

60438 Frankfurt

Germany

Telephone +49 (0)69 798 29513

Telefax +49 (0)69 798 29527

E-Mail e.boles@bio.uni-frankfurt.de

Mislav Oreb

Goethe University Frankfurt

Institute of Molecular Biosciences

Max-von-Laue Straße 9

60438 Frankfurt

Germany

Telephone +49 (0)69 798 29331

Telefax +49 (0)69 798 29527

E-Mail m.oreb@bio.uni-frankfurt.de

Supplementary Table S1 | Primers used in this study.

Primer name	Application	Sequence 5'-3'
Amp_GAL2_F	Forward primer for GAL2 amplification	ATGGCAGTTGAGGAGAAC
Amp_GAL2_R	Reverse primer for GAL2 amplification	TTATTCTAGCATGGCCTTG
Clon_GAL2_F	Forward primer for GAL2 cloning	AACACAAAAACAAAAAGTTTTTTTAATTTTAATCAAAAAATGGCAGTTGAGGAGAACAA
Clon_GAL2_R	Reverse primer for GAL2 cloning	GAATGTAAGCGTGACATAACTAATTACATGACTCGAGTTATTCTAGCATGGCCTTGTACC
SRp001	Forward primer for cloning GFP, with overhangs for fusing it to Gal2	GGTACAAGGCCATGCTAGAAATGAGTAAAGGAGAAGAACTTTTCAC
SRp002	Forward primer for cloning GFP, with overhangs for CYC1t	AACTAATTACATGACTCGAGTTATTTGTATAGTTCATCCATGCCATG
SRp014	Reverse primer for cloning GAL2, with overhangs for fusing it to GFP	AGTTCTTCTCCTTTACTCATTTCTAGCATGGCCTTGTACC
VSP66	Forward primer for mutagenesis of M435 to I in GAL2	GGTGCCGGTAACTGTATCATTGTCTTTACCTG
VSP67	Reverse primer for mutagenesis of M435 to I in GAL2	ACAGGTAAAGACAATGATACAGTTACCGGCACC
VSP31	Forward primer for mutagenesis of N376 to Y in GAL2	GTCATTGGTGTAGTCTACTTTGCCTCCACTTTCTTTAG
VSP32	Reverse primer for mutagenesis of N376 to Y in GAL2	GAAAGTGGAGGCAAAGTAGACTACACCAATGACAATGG
VSP62	Forward primer for mutagenesis of M107 to K in GAL2	TTGAGAAGGTTTGGTAAGAAACATAAGGATGGTACC
VSP63	Reverse primer for mutagenesis of M107 to K in GAL2	ACCATCCTTATGTTTCTTACCAAACCTTCTCAAAAAGTCTG
VSP64	Forward primer for mutagenesis of V239 to L in GAL2	AGCTATTCGAACTCACTTCAATGGAGAGTTCCATTAGG
VSP65	Reverse primer for mutagenesis of V239 to L in GAL2	TGGAACTCTCCATTGAAGTGAGTTCGAATAGCTCTTTG
VSP68	Forward primer for mutagenesis of L558 to S in GAL2	GGTAATAATTACGATTCAGAGGATTTACAACATGACG
VSP69	Reverse primer for mutagenesis of L558 to S in GAL2	ATGTTGTAAATCCTCTGAATCGTAATTATTACCTCTTC
SRp0171	Forward primer for deletion of GAL2 with CRISPR Cas9 (gRNA)	GCCGGTGAGTCAGGGCCTGAGTTTTAGAGCTAGAAATAGCAAGTTAAAATAAGG
SRp0172	Reverse primer for deletion of GAL2 with CRISPR Cas9 (gRNA)	CTCAGGCCCTGACTCACCGGCGATCATTTATCTTTCACTGCGGAG
SRp0157	Donor DNA for repairing GAL2 locus (forward strand)	ATGGCAGTTGAGGAGAACAATATGCCTGTTGTTTCACAGCAACCCCAAGCTGGTGAAGACGTGATCTCTTAGGTAATAATTACGATTTAGAGGATTTACAACATGACGACAAACCGTGGTACAAGGCCATGCTAGAATAA
SRp0158	Donor DNA for repairing GAL2 locus (reverse strand)	TTATTCTAGCATGGCCTTGTACCACGGTTTGTCGTCATGTTGTAAATCCTCTAAATCGTAATTATTACCTAAGAGATCACGTCTTCACCAGCTTGGGGTTGCTGTGAAACAACAGGCATATTGTTCTCCTCAACTGCCAT
SRp0201	Forward primer for verification of GAL2 deletion	ATGGCAGTTGAGGAGAACAATATG
SRp0202	Reverse primer for verification of GAL2 deletion	TTATTCTAGCATGGCCTTGTACCAC

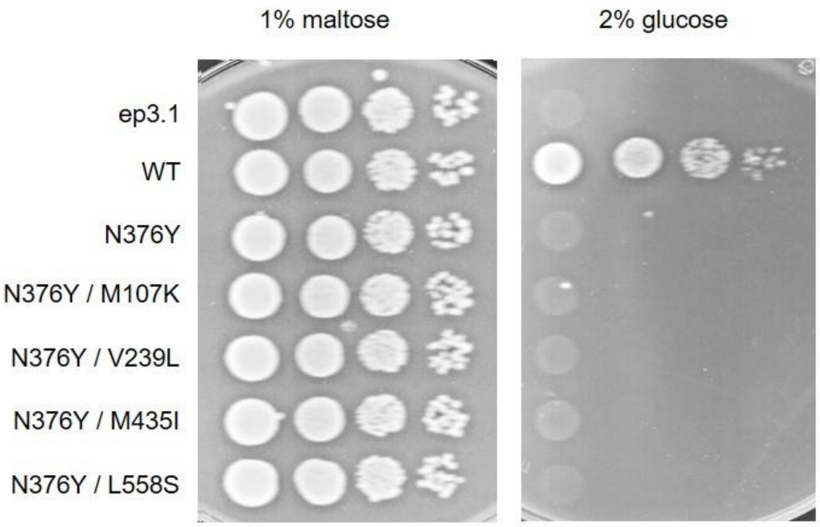

Supplementary Figure S1 | Effect of the mutations in the Gal2 clone ep3.1 on glucose transport. The hxt^0 strain EBY.VW4000 was transformed with plasmids encoding the ep3.1, wildtype (WT) or indicated variants of Gal2 generated by site directed mutagenesis. Dilution series of cell suspensions were spotted onto agar plates containing the indicated sugars (concentrations are given as % w/v) and the growth was analyzed after three days of incubation at 30°C. Maltose as a permissive carbon source for the hxt^0 strain was used to control the viability of the transformants.

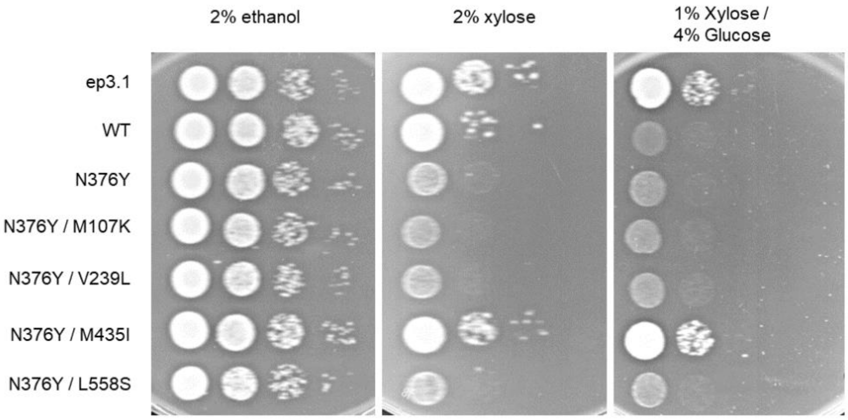

Supplementary Figure S2 | Effect of the mutations in the Gal2 clone ep3.1 on xylose transport. The hxt^0/hxk^0 strain AFY10X was transformed with plasmids encoding the ep3.1, wildtype (WT) or indicated variants of Gal2 generated by site directed mutagenesis. Dilution series of cell suspensions were spotted onto agar plates containing the indicated sugars (concentrations are given as % w/v) and the growth was analyzed after five days of incubation at 30°C. Ethanol as a permissive carbon source for the hxt^0/hxk^0 strain was used to control the viability of the transformants.

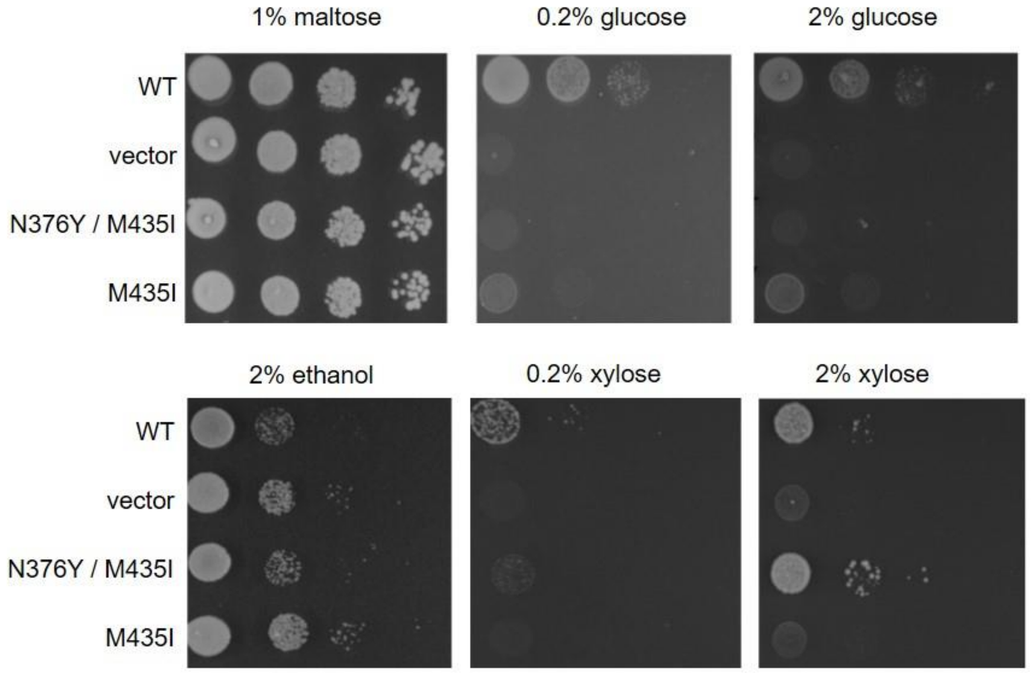

Supplementary Figure S3 | Effect of the M435I mutation alone on xylose and glucose transport. The hxt^0 strain EBY.VW4000 (top) and the hxt^0/hxk^0 strain AFY10 were transformed with plasmids encoding the indicated variants of Gal2 or the empty vector as a negative control. Dilution series of cell suspensions were spotted onto agar plates containing the indicated sugars (concentrations are given as % w/v) and the growth was analyzed after three (hxt^0 strain) or five (hxt^0/hxk^0 strain) days of incubation at 30°C. Maltose as a permissive carbon source for the hxt^0 strain and ethanol for the hxt^0/hxk^0 strain were used to control the viability of the transformants.

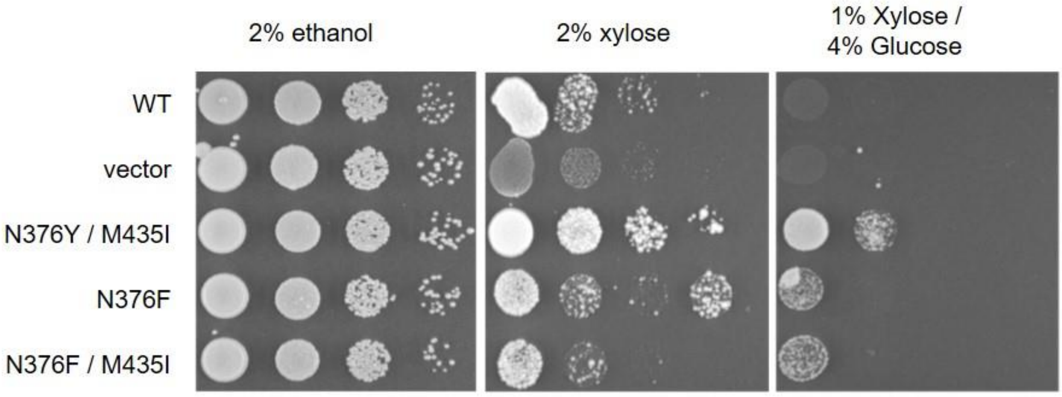

Supplementary Figure S4 | Combination of the N376F with the M435I mutation. The hxt^0/hxk^0 strain AFY10 were transformed with plasmids encoding the indicated variants of Gal2 or the empty vector as a negative control. Dilution series of cell suspensions were spotted onto agar plates containing the indicated sugars (concentrations are given as % w/v) and the growth was analyzed after five days of incubation at 30°C. Ethanol as a permissive carbon source for the hxt^0/hxk^0 strain was used to control the viability of the transformants.
